# A longitudinal comparison of maternal behaviour in German urban humans (*Homo sapiens*) and captive chimpanzees (*Pan troglodytes*)

**DOI:** 10.1038/s41598-024-51999-4

**Published:** 2024-01-17

**Authors:** Federica Amici, Katja Liebal, Manuela Ersson-Lembeck, Manfred Holodynski

**Affiliations:** 1 Faculty of Life Sciences, Institute of Biology, Human Biology & Primate Cognition, Leipzig University, Talstrasse 33, 04103 Leipzig, Germany; 2 Department of Comparative Cultural Psychology, Max Planck Institute for Evolutionary Anthropology, Leipzig, Germany; 3 Department of Education and Psychology, Comparative Developmental Psychology, Freie Universität Berlin, Habelschwerdter Allee 45, 14195 Berlin, Germany; 4 Faculty of Psychology/Sport and Exercise Studies, Institute for Psychology in Education, University of Münster, Fliednerstrasse 21, 48149 Münster, Germany

**Keywords:** Human behaviour, Animal behaviour

## Abstract

Comparative perspectives are crucial in the study of human development, yet longitudinal comparisons of humans and other primates are still relatively uncommon. Here, we combined theoretical frameworks from cross-cultural and comparative psychology, to study maternal style in 10 mother–infant pairs of German urban humans (*Homo sapiens*) and 10 mother–infant pairs of captive chimpanzees (*Pan troglodytes*), during the first year of infants’ development. We conducted focal observations of different behaviours (i.e. nursing, carrying, body contact, touching, grooming, restraining, approaching, leaving, rejection, aggression, mutual gaze, object stimulation), during natural interactions. Analyses revealed a more distal maternal style in WEIRD humans than in captive chimpanzees, with different behaviours being generally more common in one of the two species throughout development. For other behaviours (i.e. nursing), developmental trajectories differed between WEIRD humans and captive chimpanzees, although differences generally decreased through infants’ development. Overall, our study confirms functional approaches as a valid tool for comparative longitudinal studies.

## Introduction

In mammals, mothers strongly contribute to their offspring’s fitness and survival by providing them with warmth, food and protection against potential dangers^1^. In non-human primates (hereafter, primates), mothers are also crucial social partners for their offspring, providing agonistic support and opportunities for social learning, fostering their integration in the social group and their cognitive development^2,3^. In this way, immatures can gradually acquire their species-typical behaviour, and slowly grow into the social roles they will have as adults^3,4^.

### Maestripieri’s model of primate maternal styles

In primates, the most common theoretical framework for the study of mother–infant relationships is perhaps the one by Dario Maestripieri. Building on previous work in developmental psychology and on the results of a series of studies in cercopithecine monkeys, Maestripieri described different primate maternal styles, which may vary within and across species, and partially across mother’s and offspring’s age, along two independent dimensions: protectiveness and rejection^5–8^. According to this framework, protective mothers are more likely to initiate social interactions (e.g. proximity, grooming) with their offspring, as compared to less protective mothers, and they are also more likely to restrain immatures when they try to break proximity^7,9–12^. Rejecting mothers, in contrast, are more aggressive toward their offspring, and more often avoid physical contact with them, by preventing immatures from approaching or by frequently breaking contact with them^2,7,9,10,12,13^. The combination of these two independent dimensions can result in four different maternal styles: while protective mothers are highly protective (but not rejecting) and rejecting mothers are highly rejecting (but not protective), controlling mothers are both protective and rejecting, and laissez-faire mothers are rarely protective and rarely rejecting^2,11^.

The identification of these different maternal styles is important, because they appear to not only catch variation in how mothers care for their offspring, but also to effectively predict central aspects of immatures’ sociality that might be linked to these different maternal styles^2^. In macaques, for instance, mothers that are not very protective have more independent, explorative and playful offspring than highly protective mothers^13–15^. In contrast, higher rejection rates may promote offspring independence^9,13,16^, but also increase stress and depression in the offspring^17–21^. Moreover, although mothers largely maintain their maternal style across different offspring^2,10,22^, they can also adjust it to the specific needs and developmental phase of the offspring^23^. Mothers, for instance, are usually more protective towards younger offspring^24–26^. In contrast, rejecting behaviour appears to peak between the first and the second year of offspring’s development, at least in macaques^18,25,27^, when mothers start resuming their mating activities^28–30^.

These studies have provided important insight into the development of mother–infant relationships in macaques, and its role for their social development. However, studies in other primate species are scant, and direct comparisons to humans are generally hindered by this approach, because mother–infant relationships in humans typically involve a variety of other behaviours that are not exhaustively caught by this theoretical framework. In WEIRD societies (i.e. Western, educated, industrialized, rich and democratic ones^31^, for instance, mothers often engage in face-to-face interactions with their infants, and use different non-verbal means of communication, including mutual gaze, to engage in joint interactions with their offspring^32–35^.

### Keller’s component model of parenting

To effectively grasp these aspects of mother–infant relationships, and the impressive variation with which they are instantiated across human societies, Keller and colleagues proposed the component model of parenting^36–38^, which identifies six different systems of parental care, each serving a different function for the development of human infants: (i) the primary care system, which includes maternal behaviours satisfying basic needs like feeding, warming and protecting the offspring; (ii) the body contact system, which implies physical closeness and body contact, and is thought to protect infants and increase mother–infant bonding; (iii) the body stimulation system, which includes touching or moving offspring’s body or body parts, and is thought to stimulate infants’ motoric development and body perception; (iv) the face-to-face system, which implies the interaction of mothers and offspring while their faces are oriented to each other, and might facilitate dyadic and triadic interactions, and sensitivity for others’ mental states; (v) the object stimulation system, which implies the triadic interaction of mothers and offspring with objects in the environment, and might foster immatures’ explorative behaviour and cognitive development; and (vi) the narrative envelope system, which is considered a uniquely human behaviour largely based on linguistic tools, crucial for social learning processes.

Depending on how likely mothers recur to these different systems, they may show two main parenting styles: mothers with a proximal style are more likely to rely on the body contact and body stimulation systems, whereas mothers with a distal style are more likely to rely on the face-to-face and object-stimulation system^37,38^. These different parenting styles have been successfully used to describe some aspects of the huge variation in mother–infant relationships that exists across human cultural communities. In WEIRD societies, for instance, mothers often show a more distal maternal style, with frequent face-to-face interactions, eye contact and recurrent attempts to engage in different forms of joint attention^32–34^. In other societies (e.g. rural Cameroonian Nso), in contrast, proximal maternal styles are more common, as mother–infant relationships are more often characterized by body contact and tactile stimulation^32,37,39–42^. This approach, therefore, has allowed to effectively capture variation in maternal styles across human cultural communities—a crucial endeavour to document the rich variety of ways in which behaviours are instantiated across different human cultural settings.

### A comparison of both models

Clearly, both theoretical frameworks have strengths and limitations. Maestripieri’s approach, for instance, despite being yet the most important and widely used framework to study mother–infant relationships in primates, does not cover important behavioural aspects that are instead often studied in humans, like face-to-face interactions and different forms of dyadic and triadic interactions. Given that these behaviours may be less frequent or even absent in macaques, which were the main focus of Maestripieri’s initial work^5–7,11^, it is reasonable that they were not included in his framework. However, these behaviours may be more common in other primate taxa like apes, which are more closely related with humans^32,43–45^, and following their development may be highly informative to better understand the evolution of primate parenting behaviour and to identify aspects that might be unique to human parenting. Keller’s approach^36,46^, in contrast, has a strong focus on behaviours that are considered crucial for human development, but may disregard others that are important for different primate species (e.g. aggressive behaviours).

### Aims of the current study

In this study, we aimed to use both frameworks to study maternal styles in humans (*Homo sapiens*) and chimpanzees (*Pan troglodytes*). We used focal observations to assess the frequency and duration of different behaviours (i.e. nursing, carrying, body contact, touching, grooming, restraint, maternal approach, maternal leave, rejection, aggression, mutual gaze and object stimulation; see Table 1) that occurred during natural interactions in 10 mother–infant pairs of WEIRD humans and 10 mother–infant pairs of captive chimpanzees. Chimpanzees are good candidates for this comparative approach because they are—together with bonobos (*Pan paniscus*)—the closest relatives of humans, and despite important intra-specific variation, they are known to not only show basic components of maternal behaviour, but also to engage in other forms of parenting, including face-to-face interactions^32,43–45^ and perhaps triadic forms of joint attention^47^.

**Table 1:** For each maternal behaviour observed, we report (i) whether the behaviour was protective or rejecting according to Maestripieri’s^5–8,11^ framework; (ii) whether it belonged to one of the systems included in Keller’s^36,46^ framework; and (iii) the significant predictors in each model.

Model and behaviour		Maestripieri’s framework	Keller’s framework	Effect
1	Nursing (b)	–	Primary care system	Group * age
2	Carrying (p)	–	Body contact system	–
3	Body contact (p)	Protective	Body contact system	Group, age
4	Touching (p)	Protective	Body stimulation system	Group, age
5	Grooming (b)	Protective	Body stimulation system	Group
6	Restraint (b)	Protective	–	Group, age
7	Maternal approach (b)	Protective	–	Group, age
8	Maternal leave (b)	Rejecting	–	Group, age
	Rejection (b)	Rejecting	–	–
	Aggression (b)	Rejecting	–	–
9	Mutual gaze (b)	–	Face-to-face system	Group, age
10	Object stimulation (b)	–	Object stimulation system	Age

Responses were modelled as proportional responses (p) or binomial responses (b). Two terms linked by an asterisk represent an interaction. For the object stimulation system, the model only included humans, as we observed no object stimulation in chimpanzees.

Our first objective was to longitudinally compare maternal styles in WEIRD humans and captive chimpanzees during infants’ development, from 1 to 12 months of age. Although our study sample cannot clearly be considered representative of the whole human and chimpanzee species, and generalizations should be avoided^48^, the comparative approach may be useful to detect possible differences in maternal styles. Based on literature, we hypothesized that there would be differences between WEIRD humans and captive chimpanzees in maternal style for several behaviours, but that these differences between study groups were expected to decrease through infants’ development, as mothers become decreasingly important for their offspring’s survival, and frequencies and durations of most behaviours related to parenting behaviour should generally decline in line with the infant’s growing independence.

With regards to Keller’s framework^36,46^, in particular, we predicted that, through the whole first year of infants’ life, mothers in both study groups would be equally likely to engage in behaviours of the primary care system (i.e. nursing; Prediction 1), as these behaviours are necessary to satisfy infants’ basic needs and ensure their survival^36,38,46^. Furthermore, we predicted that WEIRD human mothers, by often engaging in face-to-face and joint interactions with their infants^32–34^, would generally have a more distal maternal style than captive chimpanzees, and would more likely engage in behaviours of the face-to-face system (i.e. mutual gaze) and of the object stimulation system (i.e. object stimulation), and less likely in behaviours of the body contact system (i.e. carrying and body contact; Prediction 2). Moreover, with regards to the body stimulation system, we predicted that the study groups would differ in the use of specific behaviours, with touching being overall more likely to occur in WEIRD humans than in captive chimpanzees, and grooming being more likely to occur in chimpanzees than in humans (Prediction 3), as touching and grooming may serve a similar function for the development of motor and socio-emotional skills^49–52^. Finally, with regards to Maestripieri’s framework^5–7,11^, we predicted that protective behaviours (i.e. body contact, grooming, restraint, maternal approach; but not touching, see above) would be overall more likely in captive chimpanzees than in WEIRD humans (Prediction 4), whereas rejecting behaviours (i.e. maternal leave, rejection, aggression) would be more likely in humans than in chimpanzees (Prediction 5), as infants in chimpanzee groups are probably exposed to more potential risks than infants in WEIRD human families (e.g., aggressive interactions with physical contact by other group members or attempts of other group members, e.g. young siblings, to interact with the infant in goofy ways), and chimpanzee mothers may thus be more protective and less rejecting^10,53^.

Our second objective was to assess how effectively the two theoretical frameworks allowed detecting variation in maternal style within and between the two study groups. Comparing the effectiveness of the two approaches in humans and other primates is a crucial first step to critically discuss methods that should ideally also allow reliable comparisons across species and groups. We predicted that both theoretical approaches would allow detecting variation in maternal styles in WEIRD humans and captive chimpanzees, but that Maestripieri’s approach^5–7,11^ would be more successful to catch variation in captive chimpanzees, as it was originally designed for primates, whereas Keller’s approach^36,46^ would be more effective in humans, as it was originally designed to assess cross-cultural variation in human parenting styles (Prediction 6).

## Material and methods

### Participants

We longitudinally followed 10 mother–infant pairs of WEIRD urban humans (*Homo sapiens*) and 10 mother–infant pairs of captive chimpanzees (*Pan troglodytes*), including the same number of female and male infants in both species (see Table 2 for the complete list of study subjects). We recruited human pairs from two large German cities (Leipzig and Berlin) through the participant pool of the Excellence cluster “Languages of Emotion” and study advertisements at the Freie Universität Berlin. Mothers were recruited when they were pregnant in their third trimester or when their infants were younger than 1 month, and only if they had had a full-time delivery and had no signs of post-natal depression. We informed mothers about the study procedures and asked for their written consent to participate in the study. We pseudo-anonymized the data collected on humans, and stored them on a secure server at Freie Universität Berlin, only allowing access to the data to project members.

**Table 2:** For WEIRD humans and captive chimpanzees, identity and sex of the infants observed, and individual observational effort for each mother–infant pair at 1, 6 and 12 months of infants’ age.

Study group	Subject	Sex	Observational effort (in minutes)		
			1 month	6 months	12 months
Humans	An	Male	60	60	60
	Hd	Female	61	60	60
	Hl	Female	62	60	60
	Hn	Female	61	34	65
	Jl	Female	60	60	60
	Jr	Male	64	60	60
	Ld	Male	60	60	60
	Ls	Male	64	61	60
	Mn	Female	61	60	60
	Pp	Male	60	60	60
Chimpanzees	Azibo	Male	15	28	31
	Bangolo	Male	25	15	15
	Kara	Female	183	181	183
	Kofi	Male	183	185	183
	Lobo	Male	62	46	42
	Lome	Male	59	42	28
	Mora	Female	186	182	0
	Nayla	Female	181	181	0
	Tai	Female	34	45	43
	Yara	Female	189	181	180

Captive chimpanzees were observed at the Leipzig Zoo, at the Osnabrück Zoo and at the Kristiansand Dyrepark. All chimpanzees lived in social groups, in facilities that included indoor and outdoor areas, with climbing materials and other enrichment objects. In our study, we included a limited number of mother–infant pairs due to the challenge of including more chimpanzee infants of the right age and to the fact that, for modelling purposes it was convenient to have a similar number of pairs across species. Although infants in chimpanzees and humans were matched for age, as mothers are expected to adjust their behaviour to the infants’ age, we could not match mothers’ age or parity across species (due to obvious differences in their life history traits, including age of first reproduction).

### Ethics statement

All methods were carried out in accordance with relevant guidelines and regulations. Research on the human sample was conducted according to the ethical standards of the Deutsche Gesellschaft für Psychologie (DGPs; German Psychological Association) and the ethical guidelines of the research institution (Freie Universität Berlin), as approved by the department of Comparative Psychology under supervision of Prof. Dr. Katja Liebal and the graduate school of the cluster ‘Languages of Emotion’. With regards to the chimpanzees, as our study was purely observational and required no changes in the daily routines of the individuals, it was approved by the Leipzig Zoo, the Osnabrück Zoo and the Kristiansand Dyrepark, but did not require a specific ethical approval from other institutions. The zoos complied with the WAZA Code of ethics and animal welfare^54^ and the EAZA Minimum Standards for the Accommodation and Care of Animals in Zoos and Aquaria^55^. Procedures were conform to Directive 2010/63/EU. The Office of the Ethics Advisory Board at the University of Leipzig confirmed that formal ethical approval was not required in this case.

### Behavioral observations

We used focal animal sampling^56^ to conduct behavioural observations, which we video-recorded with a digital video camera (Panasonic, HDC-HS30). We conducted observations when the infants were likely to be most active, and required no changes in participants’ daily routines. We observed humans and chimpanzees when infants were 1, 6 and 12 months old, because these ages correspond to major developmental milestones for human infants (e.g. they start social engagement at 1, they have already established a strong relationship with mothers and engage in exploratory behaviour at 6, and they already master joint attention and start engaging in cooperative behaviours at 12).

We observed human infants during home visits in three sessions (except for one infant), when they were with their mother. The father could also be present, but he was usually in another room. We observed chimpanzees during the official opening hours of the zoos, remaining in the visitors’ area to avoid changes in animals’ behaviour. As individuals lived with other conspecifics and were not separated during the study, we observed them in the presence of other conspecifics beyond the mother. For chimpanzees, the duration of the videos could vary from 5 to 60 min, so that we required several sessions for each study subject and age point. Due to the death of one chimpanzee (Mora) and the low availability of some infant chimpanzees, we could not ensure an identical observational effort across individuals and age points (Table 2).

### Coding

From the videos, we coded the following non-mutually exclusive behaviours, which have been considered protective or rejecting by Maestripieri^5–7,11^, and/or belonging to one of Keller’s parenting systems^36,46^ (see Table 1). In line with the component model of parenting by Keller^36,46^, we coded the following behaviours (see Table 1) that belonged to: (i) the primary care system: exact time the mother spent nursing the infant (i.e. the infant held the mother’s nipple in the mouth); (ii) the body contact system: exact time the mother spent carrying the infant (i.e. the mother moved while holding the infant in body contact, so that the weight of the infant’s body was supported by the mother, regardless of the mother using limbs to this purpose), and exact time spent in body contact with the infant (i.e. one mother’s body part was in contact with any body part of the infant); (iii) the body stimulations system: exact time the mother spent touching the infant (i.e. the mother placed the palm of her hand in contact with any body part of the infant), and exact time spent grooming the infant (i.e. cleaning the infant’s skin or fur by manipulating it with her hands or mouth); (iv) the face-to-face system: exact time the mother engaged in mutual gaze with the infant (i.e. the mother looked at the infant while the infant looked at her, with their faces aligned); and (v) the object stimulation system: exact time the mother engaged in object stimulation with the infant (i.e. the mother tried to attract the infant’s attention toward an object).

Following the categories used by Maestripieri^5–7,11^ (see Table 1), we further coded whether the mother (viii) restrained the infant (i.e. the mother prevented the infant from breaking body contact with her), (ix) approached the infant (i.e. the mother moved toward the infant, within one arm’s distance), (x) left the infant (i.e. the mother moved away from the infant, further than one arm’s distance), (xi) rejected the infant (i.e. the mother prevented the infant from making body contact with her), and (xii) was aggressive toward the infant (i.e. the mother showed contact or non-contact aggressive behaviours toward the infant). Finally, we coded the exact duration of each video and the exact time the mother and her infant were out of view, and considered their difference as a measure of observational effort. Please note that this approach is slightly different from the original one used by Keller and colleagues^37^.

When coding the different behaviours, Keller and colleagues^37^ divided observations into intervals of 10 s and then coded, for each interval, whether system-typical behaviours occurred for at least 5 s (e.g. body contact, face-to-face interaction) or at least once (e.g. body stimulation, object stimulation). However, this approach may be problematic, because it inflates the number of data points by considering the contiguous 10-s intervals as independent observations. A methodological safer approach is to simply code, for each focal observation, the exact duration of continuous behaviours (e.g. body contact) or the number of point events occurred (e.g. rejections). It should also be noted that we provided no evaluation of the narrative envelope system, as this is not present in non-linguistic animals like primates. Finally, we have opted to include grooming in the body stimulation and not in the body contact system (as originally proposed by Keller and colleagues^38^), because grooming in primates also covers a variety of functions beyond the ones of the body contact system^49,50,52^. In particular, grooming in primates is considered to be functionally very similar to touching (which is part of the body stimulation system in humans^38^), and both are essential for the healthy physical and emotional development of primates^50,52^.

### Statistical analyses

We conducted our statistical analyses in R (version 4.0.2^57^), using the “glmmTMB” package^58^ to run generalized linear mixed models (GLMM^59^). We conducted ten different models (M) to assess how maternal behaviours varied in the two study groups across development. We first modelled durations (i.e. nursing: M1, carrying: M2, body contact: M3, touching: M4, grooming: M5, mutual gaze: M9, and object stimulation: M10) as proportions of time spent in these behaviours (out of observational effort), using a beta distribution. If the models showed overdispersion (i.e. M1, M5, M9 and M10), we re-ran the model using a binomial response (i.e. whether the mother engaged at least once in these behaviours during the video), by using a binomial distribution and including observational effort as offset term, to control for the different time individuals were observed. Similarly, we modelled all other binomial responses (i.e. maternal restraint: M6, maternal approach: M7, and maternal leave: M8) with a binomial distribution, adding observational effort as offset term. We ran no model for maternal rejection and aggression, as these behaviours were never observed in the two study groups (in contrast to what happens in both captive and wild macaques^9,13,16,18–20,60^.

In all models, the test predictor was the 2-way interaction of study group (i.e. WEIRD humans or captive chimpanzees) with developmental phase (which we also included as main effects), as this allowed us to assess if our responses varied across developmental phases in different ways for the two study groups. In all models, we also included infant identity as random factor, as each individual was observed more than once. Each full model was then compared with likelihood ratio tests to a corresponding null model that only contained offset terms and random effects, but no test predictors^61^. In case of a significant comparison, the drop1 function allowed assessing whether the 2-way interaction had a significant effect, and if not, we re-ran the model only including the terms of the interaction as main effects, and assessed their significance. Similarly, to avoid unreliable model estimates, we removed the interaction from the model and only included its terms as main effects, if the analyses revealed complete separation of data when including the interaction (M5, M8 and M9). As object stimulation was only observed in WEIRD humans at all developmental phases, we re-ran M10 after completely removing study group from the full model. We finally used the package emmeans to analyse significant interactions and categorical predictors with more than one level (i.e. developmental phase^62^). We detected no convergence issues in any of the models presented. We finally checked overdispersion (see above) and multicollinearity using the “DHARMa”^63^ and the “performance” packages^64^. In all models, multi-collinearity was low (maximum variance inflation factors across models = 1.16^65^).

## Results

### Primary care system

**Figure 1:**
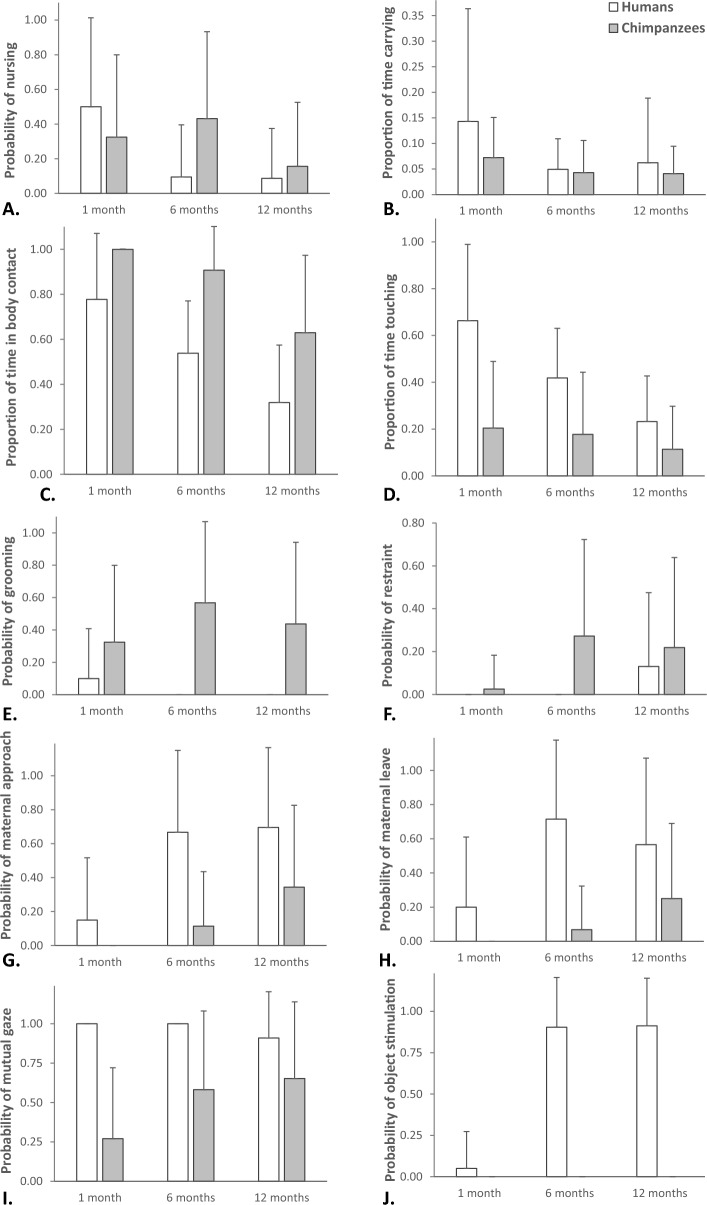
For mothers in WEIRD humans (in white) and captive chimpanzees (in grey) with infants aged 1, 6 and 12 months, average (with standard deviation) (**A**) probability of nursing infants, (**B**) proportion of time carrying, (**C**) being in body contact with or (**D**) touching infants, (**E**) probability of grooming or (**F**) restraining infants, (**G**) probability of maternal approaches or (**H**) leaves, (**I**) probability of mutual gaze or (**J**) object stimulation of infants. In this figure, proportions are calculated as in the models (i.e., as time spent in these behaviours, out of observational effort), whereas probabilities simply measure the average likelihood that a behaviour occurred for a certain species and age (whereas in the models we also accounted for differences in the observational effort by including the latter in the models as offset term).

For Model 1, the full-null comparison was significant (GLMM, *χ*^2^ = 25.12, *df* = 5, *p* < 0.001), with an effect of the interaction of study group and developmental phase on the probability of nursing (*p* = 0.005; Table 3). In particular, the probability of nursing decreased in the first year of life for WEIRD humans, with a steep decrease from 1 to 6 months, whereas for captive chimpanzees it remained relatively high through the whole first year of infants’ life, with a peak at 6 months, when it was higher than in WEIRD humans, and then decreased again (Fig. 1A).

**Table 3:** Results of the five models run, with estimates, standard errors (SE), confidence intervals (CIs), likelihood ratio tests (LRT), degrees of freedom (df), and *p* values for all test predictors (reference category in parentheses; significant *p* values marked with an asterisk).

Models	Estimate	SE	2.5% to 97.5% CIs	*LRT*	*df*	*p*
Model 1: Probability of nursing (primary care system)						
Intercept	− 14.68	0.50	− 1.65 to − 13.71	–	–	–
Group (humans) * infant’s age (6)	− 3.23	1.08	− 5.35 to − 1.11	10.74	2	0.005*
Group (humans) * infant’s age (12)	− 1.32	1.18	− 3.62 to 0.98			
Group (humans)	0.31	0.74	− 1.14 to 1.76			
Infant’s age (6)	0.81	0.55	− 0.28 to 1.89			
Infant’s age (12)	− 1.12	0.77	− 2.63 to 0.38			
Model 2: Proportion of time carrying (body contact system)						
Intercept	− 2.36	0.16	− 2.68 to − 2.04	–	–	–
Group (humans)	0.17	0.19	− 0.20 to 0.54	0.77	1	0.379
Infant’s age (6)	− 0.38	0.17	− 0.71 to − 0.05	6.63	2	0.036
Infant’s age (12)	− 0.42	0.18	− 0.77 to − 0.06			
Model 3: Proportion of time in body contact (body contact system, protective)						
Intercept	2.26	0.21	1.84 to 2.68	–	–	–
Group (humans)	− 1.42	0.23	− 1.88 to − 0.96	20.94	1	< 0.001*
Infant’s age (6)	− 0.53	0.20	− 0.92 to − 0.15	37.51	2	< 0.001*
Infant’s age (12)	− 1.41	0.22	− 1.84 to − 0.97			
Model 4: Proportion of time touching (body stimulation system, protective)						
Intercept	− 0.94	0.22	− 1.37 to − 0.50	–	–	–
Group (humans)	1.10	0.29	0.53 to 1.68	11.57	1	< 0.001*
Infant’s age (6)	− 0.34	0.19	− 0.71 to 0.04	15.11	2	< 0.001*
Infant’s age (12)	− 0.83	0.21	− 1.25 to − 0.42			
Model 5: Probability of grooming (body stimulation system, protective)						
Intercept	− 14.55	0.36	− 15.25 to − 13.84	–	–	–
Group (humans)	− 3.86	0.76	− 5.36 to − 2.36	33.89	1	< 0.001*
Infant’s age (6)	1.07	0.48	0.13 to 2.01	5.12	2	0.077
Infant’s age (12)	0.58	0.53	− 0.46 to 1.61			
Model 6: Probability of restraint (protective)						
Intercept	− 18.00	1.02	− 19.99 to − 16.00	–	–	–
Group (humans)	− 1.96	0.67	− 3.27 to − 0.66	10.93	1	< 0.001*
Infant’s age (6)	2.90	1.08	0.79 to 5.01	17.68	2	< 0.001*
Infant’s age (12)	3.16	1.09	1.01 to 5.30			
Model 7: Probability of maternal approach (protective)						
Intercept	− 19.21	1.02	− 21.22 to − 17.20	–	–	–
Group (humans)	2.41	0.78	0.89 to 3.93	9.80	1	0.002*
Infant’s age (6)	3.15	0.87	1.45 to 4.85	44.66	2	< 0.001*
Infant’s age (12)	4.39	0.94	2.55 to 6.23			
Model 8: Probability of maternal leave (rejecting)						
Intercept	− 19.21	1.01	− 21.18 to − 17.23	–	–	–
Group (humans)	2.86	0.80	1.29 to 4.42	12.94	1	< 0.001*
Infant’s age (6)	2.75	0.81	1.16 to 4.34	27.89	2	< 0.001*
Infant’s age (12)	3.44	0.87	1.73 to 5.15			
Model 9: Probability of mutual gaze (face− to− face system)						
Intercept	− 14.82	0.46	− 15.73 to − 13.91	–	–	–
Group (humans)	3.39	0.93	1.57 to 5.21	19.98	1	< 0.001*
Infant’s age (6)	1.51	0.51	0.50 to 2.51	11.26	2	0.004*
Infant’s age (12)	1.58	0.60	0.40 to 2.76			
Model 10: Probability of object stimulation (object stimulation system)						
Intercept	− 17.33	1.03	− 19.34 to − 15.32	–	–	–
Infant’s age (6)	5.46	1.28	2.95 to 7.96	54.15	2	< 0.001*
Infant’s age (12)	5.59	1.27	3.10 to 8.09

### Body contact system

For Model 2, the full model did not significantly differ from the null model (GLMM, *χ*^2^ = 7.88, *df* = 5, *p* = 0.163), suggesting that none of the predictors included in the model (i.e. neither the interaction of study group with developmental phase, nor the main terms of the interaction) reliably predicted the proportion of time mothers carried their infants (Table 3; Fig. 1B).

### Body contact system and protective behaviours

For Model 3, the full model significantly differed from the null model (GLMM, *χ*^2^ = 63.18, *df* = 5, *p* < 0.001), with group and developmental phase both having a main effect on the proportion of time spent in body contact (both *p* < 0.001; Table 3). In particular, the proportion of time spent in body contact was overall higher in captive chimpanzees than in WEIRD humans, and it overall decreased through age (Fig. 1C).

### Body stimulation system and protective behaviours

For Model 4, the full model significantly differed from the null model (GLMM, *χ*^2^ = 27.99, *df* = 5, *p* < 0.001), with group and developmental phase both having a main effect on the proportion of time mothers spent touching their infants (both *p* < 0.001; Table 3). In particular, the proportion of time spent touching infants was overall higher in WEIRD humans than in captive chimpanzees, and it overall decreased through age (Fig. 1D). For Model 5, the full-null model comparison was significant (GLMM, *χ*^2^ = 46.33, *df* = 5, *p* < 0.001), with the probability of grooming being overall higher in captive chimpanzees than in WEIRD humans (*p* < 0.001), and grooming being never observed in WEIRD humans at 6 and 12 months (Table 3; Fig. 1E).

### Other protective behaviours

For Model 6, the full model significantly differed from the null model (GLMM, *χ*^2^ = 31.44, *df* = 5, *p* < 0.001), with group and developmental phase having both a main effect on the probability of maternal restraint (both *p* < 0.001; Table 3). In particular, the probability of maternal restraint was overall higher in captive chimpanzees than in WEIRD humans, and it overall increased through age (Fig. 1F). Finally, for Model 7, the full-null model comparison was significant (GLMM, *χ*^2^ = 60.03, *df* = 5, *p* < 0.001), with group (*p* = 0.002) and developmental phase (*p* < 0.001) both having a main effect on the probability of maternal approaches (Table 3). In particular, the probability of maternal approaches was overall higher for WEIRD humans than for captive chimpanzees, and it overall increased through age (Fig. 1G).

### Rejecting behaviours

For Model 8, the full model significantly differed from the null model (GLMM, *χ*^2^ = 50.37, *df* = 5, *p* < 0.001), with group and developmental phase having both a main effect on the probability of maternal leaves (both *p* < 0.001; Table 3), and very few maternal leaves occurring in chimpanzees. In particular, the probability of maternal leaves was overall higher in WEIRD humans than in captive chimpanzees, and it overall increased with age (Fig. 1H). In contrast, we never observed rejecting or aggressive behaviours toward the infant in the two study groups, at any developmental phase.

### Face to face system

For Model 9, the full model significantly differed from the null model (GLMM, *χ*^2^ = 37.23, *df* = 5, *p* < 0.001), with group (*p* < 0.001) and developmental phase (*p* = 0.004) having both a main effect on the probability of mutual gazes (Table 3). In particular, the probability of mutual gazes was overall higher in humans than in chimpanzees, always occurring in videos of WEIRD humans at 1 and 6 months, and it overall increased with age (Fig. 1I).

### Object stimulation system

Finally, for Model 10, the full-null model comparison was significant (GLMM, *χ*^2^ = 101.79, *df* = 5, *p* < 0.001), with object stimulation never occurring in captive chimpanzees. In WEIRD humans, we found an effect of developmental phase on the probability of object stimulation (*p* < 0.001), which rapidly increased from 1 to 6 months, and then remained similarly high at 12 months of age (Table 3; Fig. 1J).

## Discussion

Our study showed that most maternal behaviours were generally more common in one of the two species (i.e. body contact, grooming and maternal restraint were overall more likely in chimpanzee mothers, whereas touching, maternal approach and leaves, mutual gaze and object stimulation were overall more likely in human mothers; see Table 1). For other behaviours (i.e. nursing), the developmental trajectories were different between WEIRD humans and captive chimpanzees, changing through time in a different way in the two species. In particular, nursing peaked at 6 months of age and remained relatively high through the whole first year of infants’ life in captive chimpanzees, whereas in WEIRD humans nursing strongly decreased from 1 to 6 months. However, these differences decreased through infants’ development, in line with our hypothesis that differences in maternal style would gradually decline through immatures’ development.

In contrast to our predictions (Prediction 1), behaviours of the primary care system followed different developmental trajectories in WEIRD humans and captive chimpanzees, with nursing strongly decreasing in humans from 1 to 6 months, but more gradually decreasing in chimpanzees after the first 6 months of life (Fig. 1A). Nursing is necessary to satisfy infants’ basic needs and ensure their survival. In humans, breastfeeding is still the main source of immunity for infants^66^, and it strongly decreases the risk of infectious morbidity and other diseases for mothers and offspring^67^. For some authors, exclusive breastfeeding for the first 6 months of infants’ life and continued breastfeeding over the first year could avoid 13% of infants’ deaths^68^. Moreover, breastfeeding fosters infants’ growth^69^, socio-cognitive development^70^ and intelligence^71^. Therefore, we expected it to be common throughout the first year of infants’ development in both study groups, slowly decreasing only after the first semester of infants’ life^36,38,46^. Nonetheless, the probability that human mothers nursed 6-month-olds in our study was around one fifth of the probability of nursing 1-month-olds (whereas nursing in chimpanzees even slightly increased from 1 to 6 months). In WEIRD humans, clearly, the availability of several alternative and/or complimentary nutritional sources may reduce the probability of nursing infants beyond the very first months, although this may clearly be different in other cultural settings.

Largely in line with our predictions (Prediction 2), captive chimpanzees showed a more proximal maternal style than WEIRD humans, more often engaging in behaviours of the body contact system and less likely engaging in behaviours of the face-to-face system and of the object stimulation system. Although we found no differences between study groups in the proportion of time mothers carried their infants (Fig. 1B), captive chimpanzee mothers were more often in body contact with their offspring (Fig. 1C) and less likely to engage in mutual gaze (Fig. 1I) and object stimulation (Fig. 1J), as compared to WEIRD humans. Object stimulation, in particular, was observed only in humans, although the situational context of the observed chimpanzees also provided objects that their mothers could have used for object stimulation. Additionally, in humans, object stimulation became more frequent from 6 months of age, perhaps because mothers anticipate the difficulties of younger infants to engage in triadic interactions. With regards to the face-to-face system, mothers in WEIRD humans always engaged in mutual gaze at least once with their 1- and 6-month-old infants, during the videos (Fig. 1I). Finer-grained analyses of our data-set on mutual gaze, which also assessed the frequency and duration of mutual gazes between mothers and infants, are largely in line with our current findings, as mutual gaze events were overall longer in WEIRD humans, and their frequency increased through age in captive chimpanzees, as in our study^43^. At first sight, our results might suggest critical differences between parenting styles between humans and chimpanzees. However, proximal maternal styles are also common in humans from other cultural communities: Cameroonian Nso farmers, for instance, show more proximal parenting styles, whereas urban-living Costa Rican middle-class children show a mixture of distal and proximal parenting styles, as compared to more distal middle-class Greek or German children^41,42^. Therefore, our results would suggest that humans share with chimpanzees an inherited bias for proximal parenting styles, although these innate tendencies might be masked in specific cultural settings, like in WEIRD communities. Therefore, these findings confirm the importance of integrating cross-cultural approaches to the comparative study of development, as WEIRD communities are often outliers in several behavioural aspects and are not necessarily representative of the whole human species^31,72,73^.

With regards to the body stimulation system, we confirmed our prediction of differences between study groups in the use of these behaviours (i.e. grooming, touching; Prediction 3). As expected, throughout infants’ development maternal grooming was more likely in captive chimpanzees than in WEIRD humans, where it was only observed at 1 month of age (Fig. 1E). In contrast, touching was common in both study groups, especially in the first months, but it was overall more likely in humans than in chimpanzees (Fig. 1D). According to Keller^36,46^, touching mainly contributes to infants’ physical development, but it also implies physical contact, and it might thus be an important way for WEIRD human mothers to maintain contact with their infants. In captive chimpanzees, in contrast, mothers are often in body contact with their infants, and touching may not be as needed. Comparisons with humans in other communities would thus be especially valuable, to see if touching also decreases in humans when body contact is more common. Moreover, grooming in primates serves a variety of functions that cover several of the parenting systems proposed by Keller^36,46^, as it reduces stress and provides security and trust (as for the primary care system), but it also contributes to mother–infant bonding and social integration (as for the body contact system), and to the physical development of primates (as for the body stimulation system). Therefore, its inclusion in any of these three systems is not straightforward. What is relevant, here, however, is that functional approaches like the one by Keller^36,46^ may be valuable tools for comparative studies to infer functional similarities across different behaviours. In our case, developmental trajectories were not identical between grooming and touching (i.e., across species, touching decreased through development, whereas grooming did not significantly vary through infants’ development), so it is not clear whether these behaviours really serve the same function, as suggested by other authors^49–52^In our study, captive chimpanzee mothers were more likely than WEIRD human mothers to be in body contact with their infants (Fig. 1C), groom (Fig. 1E) and restrain them (Fig. 1F). Only in chimpanzee groups, for instance, mothers often restrained their infants when other immatures approached them and tried to carry the infants away, often following the infants and vocalizing in a highly distressed way when immatures managed to carry the infants away. However, chimpanzee mothers were also less likely than human mothers to touch (Fig. 1D) and approach (Fig. 1G) their offspring. Therefore, our results provide no clear support to the prediction that captive chimpanzees would be more likely to show protective behaviours, as compared to WEIRD humans (Prediction 4). In the same line, we found no clear support to our prediction that rejecting behaviours would be more likely in humans than in chimpanzees (Prediction 5). Although maternal leaves were more likely in humans (Fig. 1H), indeed, we found no differences between study groups in terms of maternal rejecting or aggressive behaviours, which we never observed at any developmental phase. Therefore, although infants in WEIRD human families are likely exposed to less potential risks than infant chimpanzees, this resulted in no clear differences in how protective or rejecting mothers were, except for the fact that WEIRD human mothers were more likely to leave their offspring as compared to captive chimpanzee mothers. These results suggest that Maestripieri’s framework^5–7,11^, which has been successfully used to study maternal style in several primate species, might not be easily generalized to the study of maternal style in humans and/or species like chimpanzees, where the probability of certain behaviours (e.g. aggressive and rejecting ones) toward infants may be very low (although it cannot be excluded that humans might behave differently in the absence of a human observer as it was the case in our study). These findings also suggest that there might be substantial differences between apes (including humans) and other primates in some crucial aspects of their maternal styles, with aggressive and rejecting behaviours being commonly used only in species other than apes. Moreover, the same behaviours might have a different function in humans and other primate species: behaviours that have a protective function in Cercopithecines (e.g. mothers approach the offspring to deter or displace aggressors), for instance, might have acquired a completely different function in humans (e.g. mothers approach the offspring to provide comfort and reassurance to distressed offspring). In this respect, again, a functional approach may be especially useful for comparative research.

Finally, we predicted that both theoretical approaches would allow detecting variation in maternal styles in WEIRD humans and captive chimpanzees, but that Maestripieri’s approach^5–7,11^ would be more successful to catch variation in chimpanzees, whereas Keller’s approach^36,46^ would be more effective in humans (Prediction 6). Within Maestripieri’s framework^5–7,11^, we observed five protective and three rejecting behaviours, and we found little variation for two protective behaviours (i.e. grooming and restraint, which were very seldom in humans) and two rejecting behaviours (i.e. rejection and aggression, which were completely absent in both study groups). Within Keller’s framework^36,46^, in contrast, we observed seven different behaviours across five parenting systems, and for most of them we detected variation across study groups and developmental phases, with the exception of grooming (which was very rare in humans) and object stimulation (which was absent in chimpanzees). Therefore, we found little support to our prediction (Prediction 6): Maestripieri’s framework^5–7,11^ better caught variation in protective behaviours in chimpanzees, but most rejecting behaviours were not observed in neither study group. In contrast, Keller’s framework^36,46^ appeared to be an interesting alternative approach to the comparative study of maternal styles in humans and apes, by allowing the detection of most behaviours in humans and chimpanzees at different developmental phases, except for grooming and object stimulation, which appeared to be common only in chimpanzees and humans, respectively. Although other studies have occasionally reported object stimulation and triadic interactions between chimpanzee mothers and infants^74–76^, these behaviours are not frequent in apes, even in the presence of abundant natural objects in the environment (e.g., stones, sticks, leaves), like in our study.

Our study has several important limitations. First, it included a very small sample size, which cannot capture the rich variation with which maternal behaviours can be instantiated across different individuals and settings. In humans, for instance, there is substantial variation in maternal styles within and across cultural settings^37,77^. Moreover, also within chimpanzees there is important inter-individual and inter-group variation, as their maternal style might be affected by their previous experiences and by the social dynamics of the group in which they live^45,78^. To capture this intra-specific variation, therefore, future studies should ideally include humans from other communities and/or socio-cultural contexts, and a larger number of chimpanzees from different settings, including wild ones, which will be essential to ensure the generalizability of our results and control for other factors that might also affect maternal behaviour (e.g., mothers’ parity and previous experience, presence of siblings). Second, both WEIRD humans and captive chimpanzees were observed when infants were 1, 6 and 12 months old, because these ages represent major developmental milestones for human infants. However, these ages may correspond to different developmental phases for chimpanzees^38^. In the future, it will be essential to systematically identify developmental milestones in chimpanzees and other primates, and better match them with those of humans (for a first step in this direction^79^). Longer observational efforts will also be especially interesting, to assess further changes in maternal styles through immatures’ development.

Overall, our study provides novel information about maternal styles in WEIRD humans and captive chimpanzees. WEIRD humans generally had a more distal style than captive chimpanzees, with different behaviours being more common in one of the two species throughout development. This study also provides a first comparative assessment of two theoretical frameworks for the study of maternal styles^5–7,11,36,46^. Both theoretical frameworks allowed detecting variation in maternal style in chimpanzees and humans, although Maestripieri’s approach^5–7,11^ focuses on behaviours that may not be as relevant for humans and other apes, as compared to macaques and other monkeys.

## Data Availability

Data will be made available upon request to the last author.
